# 

**Published:** 1892-11

**Authors:** 


					﻿Vol Xli.
NOVEMBER, 1802.
NO. 117
THE
Homeopathic phy^iciah,
A Monthly Journal of Homoeopathic Materia Medica
and Clinical Medicine.
*• He is a freeman whom truth makes free, and all are slaves beside."
"Seek the Truth: come whence it may, cost uhat it will."
EDITED ARD FI’BUBBED BT
WALTER M. JAMES, N4. D.
PHILADELPHIA :
No. 1125 SPRUCE 8TKKKT.
■E.jb	>
ALFRED HEATH A CO., Sole Agemts for Great Britain,
114 Ebury Street, Eaton Square.
49*yearly Subscription, $9.60. invariably in advance. Single numbers, 96 cts,
Inured at the Philadelphia PesVOffioe as BeoeoJ Claai Mall Matter.
				

